# Supplementary material for: Modeling surface color discrimination under different lighting environments using image chromatic statistics and convolutional neural networks
Source: J Opt Soc Am A Opt Image Sci Vis. 2023 Feb 15;40(3):A149–59. doi: 10.1364/JOSAA.479986 (PMC7614229; doi:10.1364/JOSAA.479986)
Supplement: Supplementary file 1 [file josaa-40-3-A149-s001.pdf]

Supplemental Document

Journal of the Optical Society of America A
OPTICS, IMAGE SCIENCE, AND VISION

Modeling surface color discrimination under different lighting environments using image chromatic statistics and convolutional neural networks: supplement

SAMUEL PONTING,[1,†] TAKUMA MORIMOTO,[1,2,†,*] AND HANNAH E. SMITHSON[1]

[1] *Department of Experimental Psychology, University of Oxford, Oxford, UK*
[2] *Department of Psychology, Justus-Liebig-Universitat-Giessen, Giessen, Germany*
[†] *These authors contributed equally to this paper.*
[*] *Corresponding author:* takuma.morimoto@psy.ox.ac.uk

This supplement published with Optica Publishing Group on 15 February 2023 by The Authors under the terms of the Creative Commons Attribution 4.0 License in the format provided by the authors and unedited. Further distribution of this work must maintain attribution to the author(s) and the published article's title, journal citation, and DOI.

Supplement DOI: https://doi.org/10.6084/m9.figshare.21896625

Parent Article DOI: https://doi.org/10.1364/JOSAA.479986

Supplementary material

Figure S1 shows RMSE for all tested models in this study. Generally speaking, image segmentation models shown by light green symbols perform well in environments 1 and 3 while for environment 2, 1-D chromatic statistics models are the best predictors though its advantage is marginal.

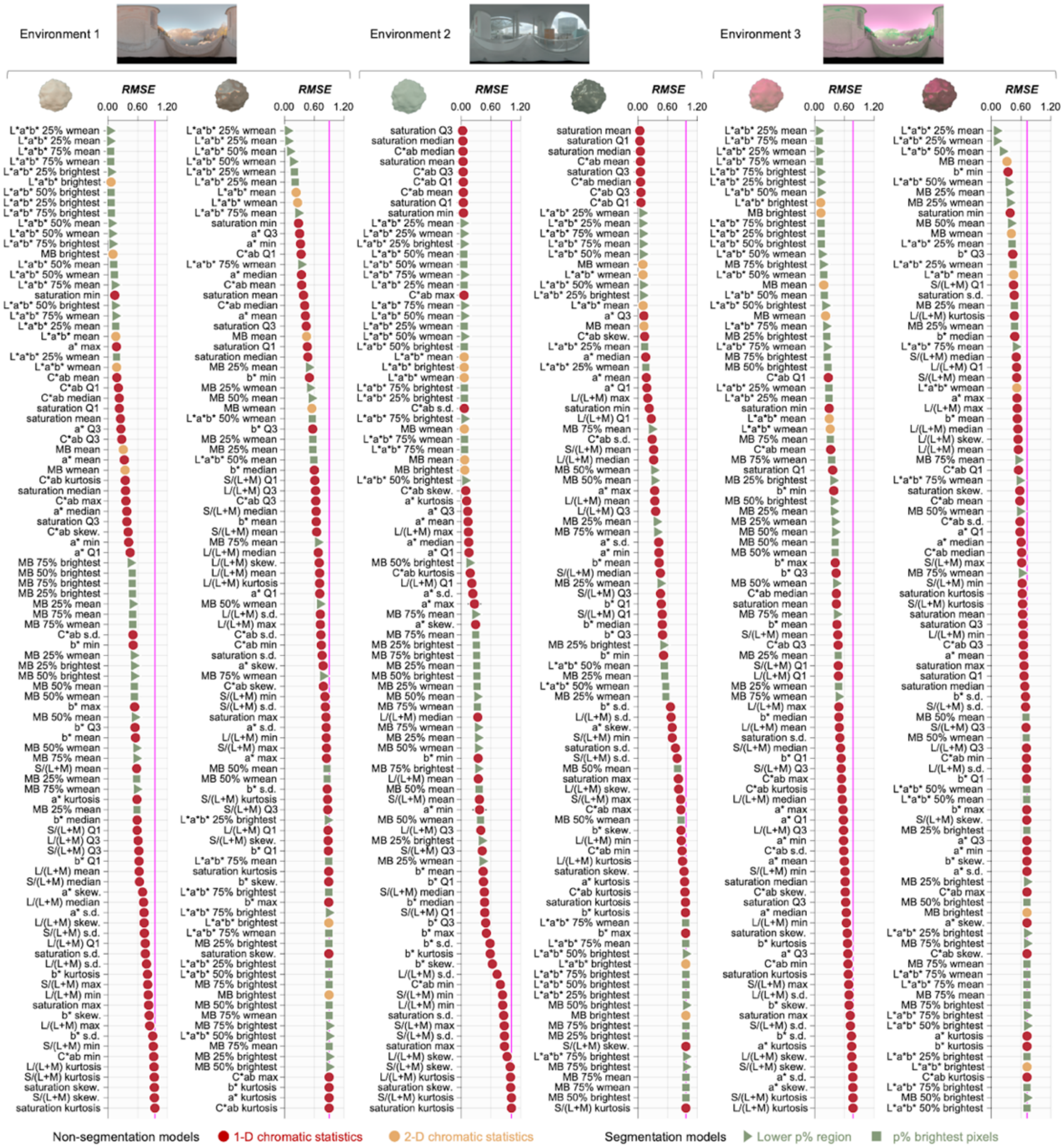

Figure S1: Performance of all chromatic statistics models tested in this study sorted by RMSE values in predicting human performance. Vertical magenta lines show models whose chromatic thresholds could not be estimated for any hue direction because discrimination thresholds fell beyond the gamut defined by realizable natural reflectance samples.
